# Lectures on Scarlet Fever

**Published:** 1851-02

**Authors:** Caspar Morris

**Affiliations:** Philadelphia, late Lecturer on Practical Medicine in the Philadelphia Medical Institute


### MEDICAL EXAMINER,

AND

### RECORD OF MEDICAL SCIENCE.

NEW SERIES.—NO. LXXIV.—FEBRUARY, 1851.

---

### ORIGINAL COMMUNICATIONS.

---

*Lectures on Scarlet Fever.* By CASPAR MORRIS, M. D., Philadelphia, late Lecturer on Practical Medicine in the Philadelphia Medical Institute.

### LECTURE I.

*Description of the Disease.*

So fatal have been the results, so fearful the ravages, so wide spread the devastation of this disease, so interesting the period of life at which it commonly occurs, just as parental hopes are budding with promise, and the tendrils of affection entwining themselves the most closely round the heart, that the very name is a signal of distress, and its introduction into the family circle is looked upon as the entrance of the angel of death with an irreprievable warrant to destroy. The parent sits down in the evening the happy centre of a group of smiling objects of affection, his heart swelling with delightful anticipation as his eye glances around the circle; and ere the next return of the same weekly period, half of them slumber in the embrace of death. The mother's nightly visit is paid to the couch of the only idol, upon whom her hopes are concentrated, and to which the sacrifice of anxious love is hourly offered, and leaves it sleeping tranquilly, no forecast shadow giving sign of the coming sorrow; the morning finds it stricken down by disease, and the evening an inanimate corpse given over to the power of corruption. These are no fancies, no exaggerated images of wo. There is not a practitioner of any extended experience but must acknowledge that he has not only witnessed such scenes, but been a miserable participator in their anxieties and grief. Of many such instances it may be said with truth, no skill could avert the result. Alas! of too many, that well meant but ill directed effort—to use the language of Sydenham, "nimia diligentia medici"—has hurried them needlessly to the tomb.

Nor is it by this rapidity of its course, alone, that scarlet fever is invested with its character of dread. Treacherous beyond all other diseases, cases apparently the least violent, "scarcely deserving the name of disease" in the earlier stages, not unfrequently develop lesions of the most formidable kind in their progress, and result in a fatal termination; while in others, a train of painful and disgusting sequela gradually exhaust the strength with long continued suffering, and entail consequences which remain for years, or even during life. It must be evident that such a disease demands the most careful investigation into its character, cause, and treatment.

I shall not detain you from the consideration of these by any discussion of the various names by which it has been called, nor of the propriety of the divisions which have been adopted by different authors. Objections may be brought against, and arguments adduced in favor of, most if not all these terms and divisions. A name it must have, and *Scarlet Fever* will answer as well as any that can be proposed; though you will find as we progress in the consideration of its symptoms, that some cases have little and others none of the peculiar eruption from which this term is derived. Divisions we must make in order to enable you to classify the cases, and arrange properly your ideas of its character and treatment; and the terms *simple*, *anginose*, and *malignant*, will embrace most cases, though I shall be obliged to notice others, which, not being reducible to either of these classes, though evidently derived from the same origin, I shall designate as *irregular.* Never, however, allow yourselves to forget that these are but various phases of one disease, which fall into each other by imperceptible shades; so that while the cases which may be assumed as types of each class have strong marks of distinction, there are others which it is difficult to distribute to either.

There is no disease the onset of which is more unexpected or more violent. Rarely indeed are there any premonitory loss of appetite, languor, or headache. Even when the known presence of the disease in a family leads to anxious watchfulness, no deviation from the usual habits marks the individual subject of the next attack. The invasion is generally in the night. A child spends the day in the most perfect enjoyment of health, runs abroad freely, eats heartily, retires to bed as usual, sleeps soundly during the earlier hours of the night, awakes with sick stomach, vomits freely, throwing off the food taken perhaps at the mid-day meal, becomes restless and fretful, has great thirst and heated skin, and when returning daylight affords opportunity of investigation, is found thickly covered upon the face, neck, and chest with a vivid scarlet rash, consisting of minute points interspersed upon a uniform effloresence, which disappears under the pressure of the finger, but is instantly renewed on its removal. The physician is sent for and finds the pulse beating with a rapidity almost pathognomonic of the disease, so that if precluded from any other mode of investigation, he might, with but little danger of error, predicate scarlet fever from its great frequency alone. The tongue is red and slightly coated with a white fur, through which frequent villi project, reminding him of the strawberry, by contrast; the surface being white and the protruding points of a vivid scarlet color. He looks further, and sees the whole mucous membrane of the mouth and fauces partaking of the same intense redness, with stigmata thickly sown upon it. The child is restless and distressed. Such would be the symptoms of the invasion of a case of simple Scarlet Fever.

Ushered in thus suddenly, the disease pursues a rapid course. Scarcely is the existence of the rash recognized before it is extended to the whole surface of the body; and finally to the lower extremities, which it reaches by the third day, at which period it begins to fade from the parts on which it first presented itself; the whole duration of the disease not often exceeding five days. There is not in this, as in the pustular and vesicular exanthems, a diminution of the febrile symptoms with the appearance of the eruption. The rapidity of the pulse which is rarely less than 120 per minute, and the heat of the skin, remain unabated. There is no disease in which the heat of the surface is greater. Not only does it communicate a peculiar pungent sensation to the finger, but by actual measured observation it sometimes rises to 105°, 108°, or 110° of Fahrenheit. There is no elevation of the rash *essential* to this eruption, which is often attended by an itching almost as intense as that of urticaria; but in some cases there are small papular elevations on the arms and legs, and it is not at all uncommon to find innumerable clear vesicles of very minute size scattered over the thorax and abdomen, which are very perceptibly elevated both to the sight and touch. The serum they at first contain is rapidly absorbed, leaving the cuticle to form minute glistening scales, separating much sooner than the general desquamation, which takes place at the close of the disease. The intensity of the eruption does not mark the degree of violence; some very mild cases having a vivid universal rash, while in others equally benign it is partial in extent and less bright in its hue. In these cases of simple scarlet fever the condition of the digestive organs varies much. While in some there is an entire loss of appetite, in others there is as constant a desire for food as in health. The bowels are unaffected in the simple form, and indeed in the primary stage of all the varieties of the disease. The kidneys too, maintain their healthy functions during the first four days, but toward the close of many cases the urine becomes scanty in quantity and high colored, but destitute of any deposit.

The functions of the skin are impaired in proportion to the intensity of the eruption. In some mild cases I have known it to continue moist and even perspirable during the whole progress of the disease.

Peculiar as was the appearance of the tongue in the beginning, it becomes still more marked as the case advances. The fur which in the variety now under consideration is never dense, is thrown off suddenly on the second or third day, beginning generally to clear off at the tip and sides, and the surface is left more entirely denuded than in health, of a color varying in intensity in proportion to the violence of the case. Sometimes but slightly reddened, at others it assumes a hue not much differing from that of raw beef, while the papillæ, elongated or swollen, rest upon a surface often smooth and glazed. This condition of tongue is doubtless owing to the same process taking place in the mucous membrane as that which is known as desquamation of the cuticle, and continues about the same length of time. The symptoms are sometimes so mild, and the nervous system is so little involved in the disease, that the patient may pass through all the stages without requiring any attention. You will find, however, when we come to treat of the sequela that such cases are by no means devoid of interest nor free from danger. Sometimes the character of an entire epidemic is thus mild, and this must undoubtedly have been the case in that recorded by Sydenham, who does not notice the anginose symptoms, though he describes with his usual perspicuity the symptoms as we have just given them to you.

It must not, however, be supposed that all cases of simple scarlet fever are thus mild in their character. In some instances from the intensity of the cause, and in others from the peculiarity of the individual attacked, the nervous system feels more decidedly the impression; and we then have the case complicated with symptoms of a more formidable character. The vomiting or nausea, by which, as I before remarked, the disease is almost invariably ushered in, may be accompanied or followed by violent convulsions; which are often of a peculiar, tetanic character. When the impression is less severe there will be constant contractions of the muscles of the forearm, with startings of the tendons, and twitchings of the fingers; and occasionally we have coma, or even the wildest delirium, manifested by screaming and frequent starting from slumber. On the third or fourth day it is no uncommon event to have the patient complain of pain in the ear, or seized with drowsiness, either of which, after continuing twenty-four hours, will in all probability be followed by the discharge of a thin serum from the external meatus auditorius, which excoriates the neighboring skin, and gives rise to a vesicular eruption in the adjacent parts.

As the rash fades it leaves the surface covered with a dry cuticle, which separates in very thin scales successively from the face, neck, chest, arms, and legs. The density of these scales depends upon the part of the body and the violence of the eruption. They are very delicate on the face, neck, and arms, while from the hands and feet, where the cuticle is thickened by pressure, it comes off in large patches, and may even, in some instances, be brought away entire.

In the *Anginose* variety, we have the same series of events as those just described, and generally in an aggravated degree. The rash may appear even more vivid, or it may be present only in patches about the folds of the body and on the back and abdomen, or about the flexures of the larger joints, or sometimes only on the hands and feet. The nervous symptoms are more often manifested, and more violent when present. There is often very great tremor of the muscles and disposition to sleep, and when aroused the child is fretful. On looking into the throat there will be found not only the redness which has been described as marking even the cases which have no anginose character, but also a swollen condition of the tonsils and soft palate. They are sometimes œdematous from the very commencement; and corresponding with the internal disease, we find a tumor under one or both angles of the jaw, which is circumscribed and limited in size, and entirely different both in character and appearance from the diffused swelling which will be noticed hereafter. This tumor is not peculiar to scarlet fever, but is similar to that which occurs in every case of inflammation of the tonsils. The difficulty of deglutition is considerable from the very beginning, and there is very often stiffness of the muscles of the neck. The pulse has even greater frequency than was noticed before, but generally less force, yielding more before the pressure of the finger; corresponding in this respect with the general want of tone of the whole system, the frequency evidently depending not on inflammatory stimulation, but on nervous excitement. From the very commencement of the anginose affection there is an abundant secretion of viscid mucus which adheres to the surface of the tonsils and palate, and occasions much annoyance, disturbing the sleep and causing the patient often to start as though strangling; and when this condition extends to the mucous membrance lining the nostrils, and they become obstructed also, the interruption to the free access of air not only adds to the suffering, but materially increases the danger by preventing the proper oxygenation of the blood already vitiated by the influence of the morbid poison. On examining the condition of the fauces there will often be seen deposits of lymph upon the surface of the tonsils, sometimes white, at others ash-colored and even darker, the color depending upon the presence of effused blood. These exudations have been mistaken for ulcers. In general they may be easily removed either by gargle or other mechanical agency, leaving the surface of the mucous membrane beneath them like the adjacent structure. In other cases they do cover ulcerated spots. This is, however, more frequent in that aggravated form which will claim our attention hereafter. They are generally confined to the surface of the tonsils, but occasionally extend to the fauces and even to the larynx; giving rise to one of the most formidable complications that can occur. The inflammation often extends from the fauces through the eustachian tube to the membrane lining the inner ear; giving rise to intense otitis. Still more frequently the lining of the external meatus takes on ulcerative action, as mentioned when describing the simple form. In either case the pain is severe, and the ulceration destroys the soft tissues, and even invades the bony structure of the ear; causing the loss of the ossicula and leaving the patient subject to prolonged offensive discharge and permanent loss of the sense of hearing. As in the case of the simple disease the tongue generally casts off its coat about the third day, assuming a still more angry appearance than that formerly described. Not unfrequently its edge as well as the mucous membrane lining the posterior nares and fauces take on ulcerative action, and then the lymphatic glands of the neck become much enlarged and exceedingly painful and tender to the touch. I have repeatedly seen both sides swollen, with effusion into the cellular tissue to such a degree as materially to impede the respiration of the child and totally to forbid deglutition. No more pitiable object can be presented than one of these cases. The commissures of the lips so sore that they bleed at every movement of the mouth; the tongue red and polished; the mucous membrane of the fauces turgid and ulcerated; the lymphatics of the neck swollen; and the cellular tissue of the neck infiltrated with serum, till the hollow between the jaw and clavicles is entirely filled, and the skin is stretched and looks as though it were polished vellum; the head thrown back to remove the pressure from the swollen throat and to facilitate the access of air to the larynx; the nostrils distilling an acrid sanies which is puffed out with a sputtering noise at every expiration, and excoriates the lip and cheeks as it flows over them, and even abrades the cuticle from the back of the hands, instinctively applied to remove it from the nostrils, and thus afford more freedom of respiration; the corners of the eyes ulcerated also,—the wretched child lies unable to swallow; (even the smallest portion of liquid taken into the mouth being ejected through the nose,) and suffering all the pains of hanging; till the most earnest prayer of the fondest parent is for its death. Yet even from such condition we may hope for recovery.

In the anginose form the stages of the disease are as regular as in the simple variety. They are not, however, so perceptible. This is caused by the accession of these secondary sources of irritation which give rise to a fever even more intense than that which has been already described, which naturally terminates on the fifth day. In the most favorable cases the subsidence of the primary symptoms is followed by the decline of the ulceration and consequent glandular disease, and convalescence is established, until the occurrence of some of the sequelæ, which will be noticed hereafter. In other instances these secondary results do not develope themselves until after the crisis of the primary disease has been safely passed.

There is a complication which occasionally modifies this form of scarlet fever, and has given rise to some difficulty of diagnosis which it may not be improper here to refer to. Instead of the extreme symptoms just mentioned, there is only a slight ulceration of the mucous membrane of the nares, which gives rise to symptoms resembling coryza, and the whole case may be considered a cold with rash, and its true nature not being suspected in the primary stages, is first proven by the development of more formidable symptoms in the second stage; or, if the rash be better developed, there is sometimes difficulty in distinguishing the case from one of measles, as I have known highly intelligent and experienced physicians at a loss. This is more especially liable to occur in those instances where an impeded respiration gives a darker hue than natural to the eruption.

But if in thus describing the horrors of the anginose scarlet fever, the mind turns with a shudder from the delineations of memory, what shall we say of the *Malignant form?* The mere presentation of the subject to you is "infandum renovare dolorem," and you may well desire to know it only by report. Under this term it is my intention to include only those cases which have by some been regarded as a violent form of the anginose scarlet fever, leaving for the class of *irregular* cases those varieties which, though of the most grave character, have no affection of the throat.

In *Malignant* Scarlet Fever there are violent pains of the legs and arms, and the rash may make its appearance on the back of the hands and the feet before it is seen on any other part of the body. The temperature of the body varies. In those cases in which the prostration is extreme, it is cold; and the rash, if it appear at all, has a livid hue, and may assume the appearance of purpura, and even be mingled with petechiæ and vibices. In some cases the rash comes out imperfectly for a time, and then disappears, re-appearing again so late even as the seventh day. Instead of the general flush like the efflorescence of the simple scarlet fever, there are only points about the flexures of the limbs, or those parts of the body which are kept the warmest. Often it does not appear at all, the whole body being rather of a *waxy paleness;* at other times there is an intense lurid erysipelatous flush which is even occasionally of a livid hue.

The condition of the throat and brain vary also; I have seen the brain intensely excited from the very onset of the disease, though in general there is coma more or less profound. The throat is swollen so as almost to render deglutition impossible. The tonsils, uvula, and soft palate lie flaccid, looking more like portions of purple velvet than living tissue; coated with black lymph in large portions; which, slightly loosened at the edges, betray an ulcer beneath; a horrible ichor discharges from the nostrils, and the room is filled with the most offensive fetor. In many instances the powers of life are totally prostrated from the very beginning. A child will be running about in apparently perfect health one hour

"feeling its life in every limb," and manifesting its power in untiring gambols, and the next may be found with a glassy and sunken or upturned eye, feeble and frequent pulse, cold surface, great jactitation, and will sink steadily to death in despite of every effort; the sedative influence of the miasm being so intense that no reaction takes place. The disease is not in fact fully developed, and its character might be doubted were it not that the subsequent discovery of an imperfectly formed eruption about the neck, or the occurrence of other cases in the same family or neighbourhood, afford sufficient evidence of its true nature. But the chaste and accurate description of an epidemic form of the disease given by the distinguished Fothergill, nearly a century ago, can scarcely be improved upon. He says, "it comes on generally with such a giddiness of the head as commonly precedes fainting, and a chilliness or shivering like that of an ague fit; this is soon followed by great heat; and these interchangeably succeed each other during some hours, till at length the heat becomes constant and intense. The patient then complains of an acute pain in the head, of heat and soreness rather than pain in the throat, stiffness of the neck, commonly of great sickness, with vomiting or purging, or both. The face soon after looks red and swelled, the eyes inflamed and watery as in measles, with restlessness, anxiety, and faintness. The disease frequently seizes the patients in the fore part of the day. As night approaches the heat and restlessness increase, and continue till towards morning, when after a short and disturbed slumber (the only repose they often have during several nights,) a sweat breaks out, which mitigates the heat and restlessness, and gives the disease sometimes the appearance of an intermittent." (This fluctuation, even in diseases where the lesions are fixed and unchangeable, is one of the most mysterious circumstances which passes under our observation. The cause is as yet wholly unknown.) "If the mouth and throat be examined soon after the first attack, the *uvula* and *tonsils* appear swollen; and these parts, together with the velum pendulum palati, the cheeks on either side near the fauces, and as much of them and of the pharynx behind as can be seen, appear of a florid red color." (This color varies in different cases.) "The color is commonly most observable on the posterior edge of the palate in the angle above the tonsils and on the tonsils themselves. Instead of this redness, a broad spot or patch of an irregular figure and of a pale white color, is sometimes to be seen surrounded with a florid red, which whiteness commonly appears like that of the gums immediately after being pressed with the finger, or as if matter ready to be discharged was contained underneath."

"Generally, on the second day of the disease, the face, neck, breast, and hands to the fingers ends, are become of a deep erysipelatous color, with a sensible tumefaction; the fingers are frequently tinged in so remarkable a manner that from seeing them only it has not been difficult to guess at the disease. A great number of small pimples, of a color distinguishably more intense than that which surrounds them, appear on the arms and other parts. They are larger and more prominent in those parts of the same subject, where the redness is least intense; which is generally on the arms the breast and lower extremities." (In a later edition of his works, 1754, Dr. Fothergill adds a note which is perfectly characteristic of the disease.) He says, "The *redness* and *eruption* have not accompanied this disease so regularly, during the latter part of this winter, as they did in the preceding seasons; in some cases they did not appear at all; in others not till the third or fourth day; and, as I have heard, in some not till the fifth, and even later." I have already drawn your attention to this want of eruption in many cases.

"As the skin acquires this color the sickness commonly goes off, the vomiting and purging cease of themselves, and rarely continue after the first day. The appearance of the fauces continues to be the same, except that the white places become more ash-colored, and it is now discoverable, that what at first might have been taken for the superficial covering of a suppurated tumor, is really a slough, concealing an ulcer of the same dimensions. All the parts of the fauces above mentioned are liable to these ulcerations, but they generally are first discoverable in the angles above the tonsils or on the tonsils themselves; thoughthey are of ten to be seen in the arch formed by the uvula and one of the tonsils, and also on the pharynx behind, on the inside of the cheeks, and the base of the tongue, which they cover in the manner of a thick fur. Instead of these sloughs where the disorder is mild, a superficial ulcer, of an irregular figure, appears in one or more of these parts, scarce to be distinguished from the sound, but by the inequality of surface it occasions."

"The parotid glands on each side commonly swell, grow hard, and are painful to the touch;* if the disease is violent, the neck and throat are surrounded with a large œdematous tumor, sometimes extending itself to the breast, which, by straightening the fauces, increases the danger. Towards night, the heat and restlessness increase, and a delirium frequently comes on. This symptom, which appears in some even on the first night, seems to differ considerably from the like affection in other diseases. The sick commonly answer the questions put to them properly, but with an unusual quickness; they talk to themselves incoherently when left alone, and frequently betray the first tendency to this disorder by affecting too great composure." (In this circumstance it is only coincident with other malignant diseases, as yellow fever, cholera, &c.) "This, for the most part, happens to those who sleep but little, for some are comatose and stupid, and take little notice of anything that passes. In this manner they continue during two, three, or more days; they commonly grow hot and restless toward evening, which symptoms, and the delirium, increase as night comes on; a sweat more or less profuse breaks out toward morning, and from this time they are easier during some hours; a faintness only continuing, of which they frequently complain more than of the rest of their sufferings. The disease seems to have no stated period which can properly be called its 'Ακμη or height. Some grow easier from the first day of the attack, but in general the symptoms of recovery appear on the third, fourth, or fifth day, and proceed in the following manner. The redness of the skin disappears, the heat grows less, the pulse which was hitherto very quick becomes slower, the external swellings of the neck subside, (sometimes they continue to increase and suppurate,) the sloughs on the fauces are cast off, the ulcerations fill up, the patient sleeps without confusion, is composed when awake, and his appetite begins to return.

The *pulse* during the whole progress of this disease is generally very quick, frequently 120 or more in a minute; in some hard and small, in others soft and full, but without that strength and

* This is an error. The swelling is not of the Parotid, but of the Lymphatic glands under the angle of the jaw.

firmness which usually accompany equal quickness and heat in genuine inflammatory disorders."

"If a vein be opened soon after the distemper is come on, the blood generally appears of a fresh florid red; the crassamentum is rather of a lax gelatinous texture than dense or compact; the serum yellow and in large proportion.

"The *urine* is at first crude, and of a pale whey color. As the disease advances it turns yellower, as if the bile was detected in it, and soon after the patient shows any marks of recovery, it grows turbid and deposits a farinaceous sediment."

(To be continued.)

